# Evaluation of enamel roughness after orthodontic debonding and clean-up procedures using zirconia, tungsten carbide, and white stone burs: an in vitro study

**DOI:** 10.1186/s12903-023-03194-6

**Published:** 2023-07-13

**Authors:** Ahmed A. Thawaba, Nehal F. Albelasy, Amira M. Elsherbini, Ahmad M. Hafez

**Affiliations:** 1 Department of Orthodontic, Faculty of Dentistry, Mansoura University, Mansoura, Egypt; 2 Department of Oral Biology, Faculty of Dentistry, Mansoura University, Mansoura, Egypt

**Keywords:** Dental debonding, Dental enamel, Dental Polishing, Orthodontic brackets, Surface Properties

## Abstract

**Background:**

The main goal of orthodontic debonding is to restore the enamel surface as closely as possible to its pretreatment condition without iatrogenic damage. This study aimed to compare the effects of different adhesive removal burs; zirconia burs, tungsten carbide burs, and white stone burs on enamel surface roughness.

**Materials and methods:**

Total sample of 72 extracted premolars was randomly divided into three equal groups (*n* = 24) depending on the method of adhesive removal: zirconia burs (ZB); tungsten carbide burs (TC); and white stones (WS). The metal brackets were bonded using Transbond XT orthodontic adhesive (3 M Unitek, Monrovia, CA, USA) and debonded after 24 h using a debonding plier, then the ARI was assessed. The adhesive remnants were removed using the different burs and Final polishing was performed using Sof-lex discs and spirals. Thirteen samples from each group were evaluated using a Mitutoyo SJ-210 profilometer to determine average surface roughness (Ra) and three samples from each group were examined under Scanning Electron Microscopy (SEM) to determine EDI score. The evaluations were performed at three time points; before bonding (T0), after adhesive removal (T1) and after polishing (T2) and the time consumed for adhesive removal by burs was recorded in seconds. The data were analyzed statistically by ANOVA, Tukey’s test and Kruskal–Wallis H-test.

**Results:**

Kruskal–Wallis H-test showed no statistically significant difference of ARI in all studied groups (*p* = 0.845) and two-way mixed ANOVA revealed that all burs significantly increased surface roughness at T1 compared to T0 (*p* < 0.001) in all groups with the lowest Ra values were observed in the ZB group, followed by the TC group, and WS group. The fastest procedure was performed with WS, followed by ZB, then TC bur (*p* < 0.001). After polishing (T2), Ra values showed no significant difference in ZB group (*P* = 0.428) and TC group (*P* = 1.000) as compared to T0, while it was significant in WS group (*p* < 0.001).

**Conclusion:**

zirconia bur was comparable to tungsten carbide bur and can be considered as alternative to white stone which caused severe enamel damage. The polishing step created smoother surface regardless of the bur used for resin removal.

## Introduction

In the last years of the twentieth century, composites have been applied to bond orthodontic attachments to the tooth surface to perform different orthodontic mechanics. In most cases, bonding is accomplished by mechanical retention of both the composite and bonding agent to the micro pores produced by the enamel surface roughening and by the composite mechanical interlocking into the bracket base mesh [1]. The natural surface structure of enamel has a micro-roughness of 0.59 to 0.66 μm [2].

To keep the enamel’s fluoride and mineral content, efforts are undertaken to reduce the risk of the enamel surface damage following orthodontic debonding and resin removal [3]. However, extensive care during the removal procedure may lead to incomplete removal of all adhesive resin, which leads to two significant problems. The first is the probability of leaving roughened areas that might favor dental plaque formation, subsequent demineralization, and decayed lesions. The second trouble involves the discoloration of composite remnants with time, causing an unaesthetic appearance [4].

Many attempts to introduce an effective and safe way of composite remnants removal after brackets removal has resulted in the development of a wide range of instrumentation and techniques including the band removing pliers or a hand scaler, rotary instruments such as tungsten carbide burs mounted on high or low-speed handpieces, diamond finishing burs, ultrasonic applications, stones, and specialized composite finishing burs. Furthermore, novel methods involving carbon dioxide and Yttrium–Aluminum-Garnet (Er: YAG) laser application, ultraviolet light (UV) fluorescent chemicals have been introduced [5–9].

There is a huge discrepancy in the literature about the most effective clean-up procedure for adhesive removal after bracket debonding. Some studies have shown that tungsten carbide burs resulted in less enamel loss and smoother surfaces than the diamond bur [10–12], air-abrasion with alumina particles [13, 14] or fiber-reinforced composite burs [15]. In contrast, other researchers did not recommend it as it may cause excessive enamel loss and increased surface roughness as compared to other tools [8, 15–17]. It was reported that white stone bur was effective for composite remnants removal compared to other types like the ultrafine diamond bur [18]. Zirconia bur was developed by the Morelli company, according to the manufacturer, it is indicated for the removal of adhesive resin remnants in a safe way without damaging the enamel surface.

A tungsten carbide bur is a dental instrument that consists of a shank and a head. The shank is usually stainless steel and the head is tungsten carbide which is a hard and wear-resistant material. The head of the bur can vary in shapes, sizes, and flutes depending on the different applications and materials. The flutes can also vary in number, from 6 to 8 for cutting burs, 12 to 20 for finishing burs, and more than 20 for polishing burs [19]. The tungsten carbide burs are preferred for removing ductile materials like composite resins because the rotation of these burs generates high shear forces between the bur’s blades and the resin surface which causes plastic plowing of the resin [20].

White stone burs are dental rotary instruments that have a conical or round shape and made of high-quality fine-grain aluminum oxide. This is an abrasive material can be used for contouring, shaping, polishing, and finishing a composite restorations, porcelain, glass ionomer cement, abutment teeth, and enamel surfaces [21]. Many tungsten carbide burs and white stone burs have been designed for removing adhesive resin after bracket debonding, but their effect on enamel is still controversial [22]. Zirconia bur is composed of Yttrium oxide partially stabilized tetragonal zirconia [23]. According to manufacturer, the Zirconia multiblade bur has the active tip which is equipped with eighteen blades with a left or right cutting direction and indicated for removal of orthodontic adhesive residue after bracket removal, and conditioning the enamel for later finishing.

Currently, no agreed-upon protocol that can completely remove the composite remnants without the risk of enamel damage after orthodontic bracket debonding. The null hypothesis is that there no significant differences in the quality of enamel surface roughness and morphology following orthodontic deboning and adhesive removal techniques using three different burs; zirconia burs, carbide tungsten burs, and white stone burs.

## Materials and methods

The current study obtained its approval from the Research Ethics Committee of Faculty of Dentistry, Mansoura University (A13071221). The procedures were carried out following the relevant laws and regulations. The sample size was calculated using G*power version 3.0.10 (Faul, Franz, et al. 2007, 2009) [24, 25] with the effect size of 0.43, 2-tailed test, α error = 0.05, and power = 90.0%, and was found to be 24 samples in each group based on the study of Ferreira et al. [1].

### Teeth selection and preparation

This laboratory experimental study was conducted on 72 human premolars extracted for orthodontic purposes or periodontal causes and collected from the outpatient clinics of the Oral and Maxillofacial Surgery Department. The samples were examined under proper light to confirm the following inclusion criteria, a crown structure with intact buccal surfaces free from caries, visible cracks, fractures, white-spot lesions, enamel hypoplasia, restorations, and no previous bracket bonding. The samples were cleaned to remove any debris and disinfected with 0.1% thymol to kill any germs and then stored in the distilled water at room temperature until use to avoid tooth dehydration, and the solution was changed weekly to prevent bacterial growth [26].

**Fig. 1:**
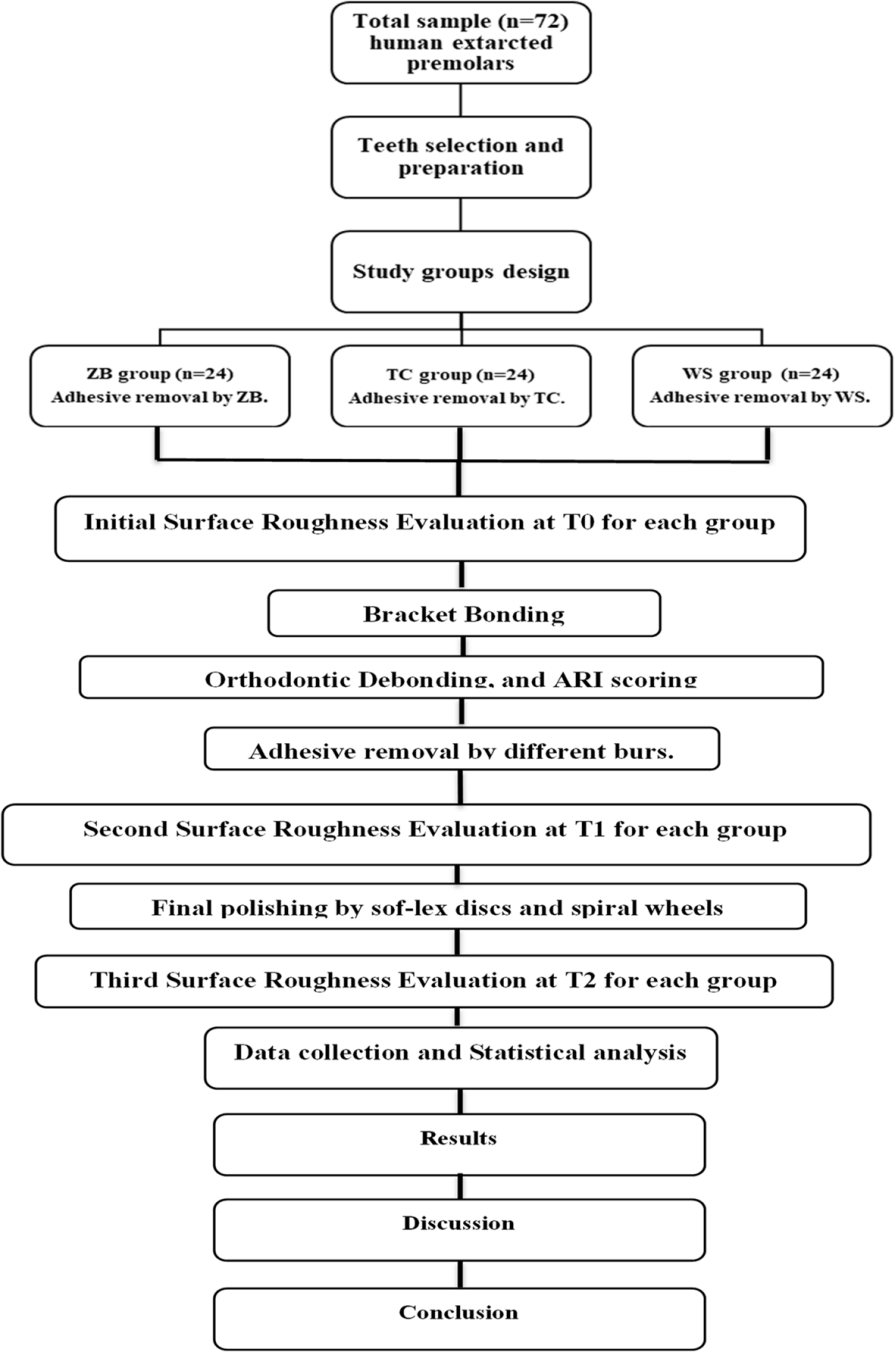
Representing the study procedures

To facilitate the handling, the root portion of each tooth was embedded in a self-cure acrylic resin block to a distance of 1–2 mm beyond the cementoenamel junction. Teeth surfaces were thoroughly cleaned and polished with a rubber cup and non-fluoridated prophylaxis paste (I-FASTE. Medicinos Linija, UAB, Lithuania). The samples were coded and numbered then the numbers were grouped randomly using https://www.random.org/ into three equal groups (*n* = 24) according to adhesive removal burs: ZB group, TC group, and WS group. Both the operator and technicians were blinded on all study steps. All study procedures were represented in Fig. 1.

### Initial surface roughness measurements

#### Profilometric analyses

**Fig. 2:**
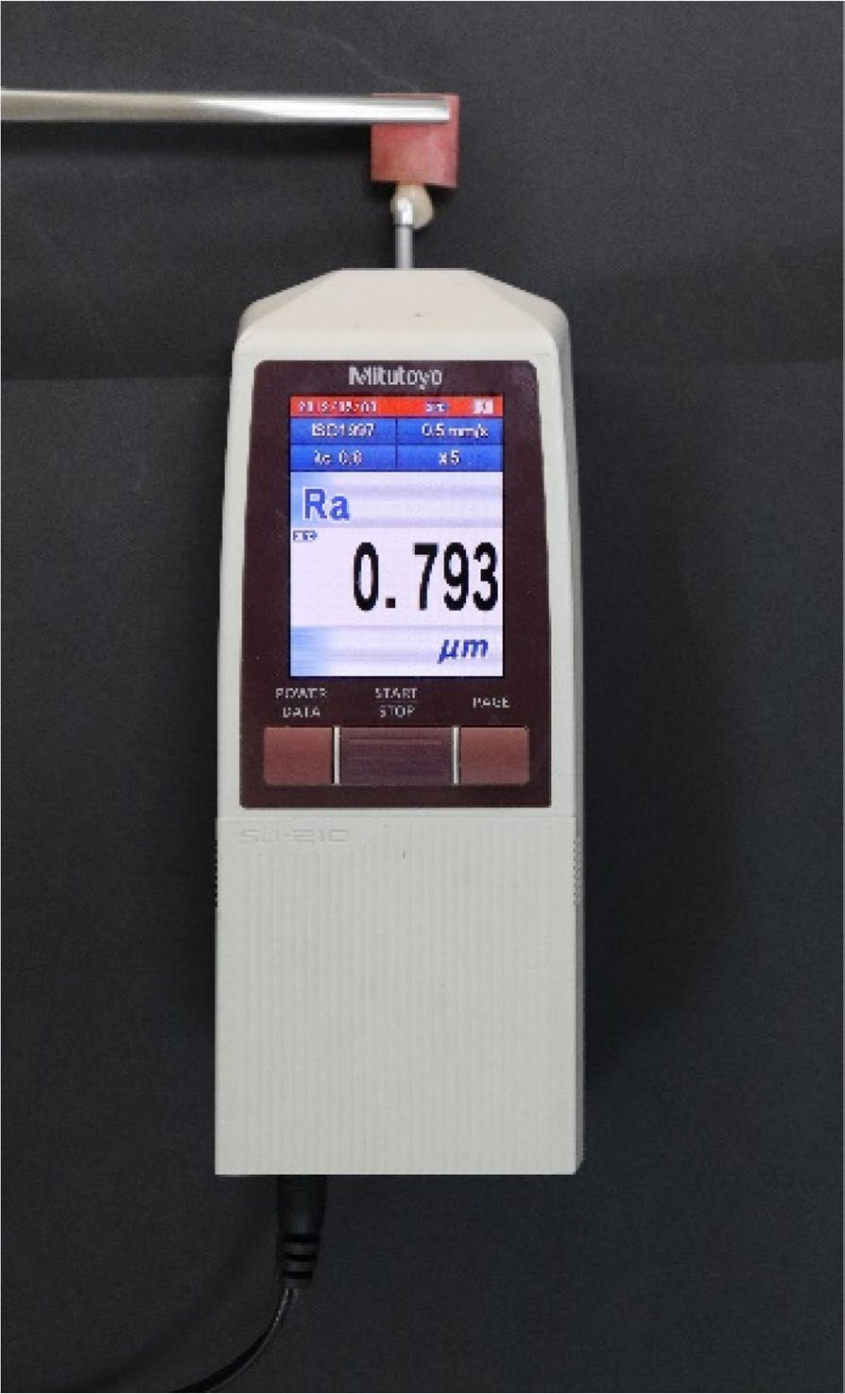
Mitutoyo SJ-210 profilometer

Before bonding (T0), Thirteen samples from each group were randomly selected using https://www.random.org/ and evaluated for Ra using a profilometer (Mitutoyo 178–560-01D Surftest SJ-21, Sakado, Japan) (Fig. 2). The measurements were performed perpendicularly on the samples at three vertical lines; the first line with the long axis of the clinical crown, the second line parallel and 0.5 mm mesial to the first line, and the third line parallel and 0.5 mm distal to the first line [27]. According to manufacturer recommendations, the stylus (tip radius of 5 μm) of the profilometer was loaded and moved on the tooth surface at a speed of 0.25 mm/second with 0.4 g force and the length of the measuring line was 0.5 mm. The average Ra values (expressed in μm) were recorded.

### Scanning electron microscopy analyses

Three teeth were randomly selected from each group using https://www.random.org/ and prepared to examine the enamel surface under SEM (Joel, JSM-6510 LV, Tokyo, Japan) before bonding (T0). The samples were dehydrated in a series of increasing ethanol concentrations (30%, 50%, 75%, 80%, 90%, 95%, and 100%) before being immersed in hexamethyldisilazane (HMDS) for 10 min [27]. Following that, the specimens were secured to stubs with double-sided resin carbon tape, sputtered with gold in a vacuum metalizing machine (SPI Module™ Sputter Coater, SPI supplies Pennsylvania, USA) for 90 s at 20 mA, and then examined with SEM at 20 to 30 kV, at a working distance ranging from 11 and 20 mm, and under magnification of 1000 X [28].

SEM images were evaluated according to the enamel damage index (EDI) developed by Schuler and Van Waes [29]. This index comprises 4 scores: 0, a smooth surface without scratches, and perikymata might be visible; 1, an acceptable surface with fine scattered scratches; 2, a rough surface with numerous coarse scratches or slight grooves visible; 3, a surface with coarse scratches, wide grooves, and enamel damage visible to the naked eye.

### Bonding and debonding procedures

The buccal surface of all teeth was etched for 15 s, with 32% phosphoric acid gel (3 M Scotchbond™ universal Etchant, USA) that was applied to the center of the buccal surface of the clinical crown, corresponding to the size of the bracket base, then rinsed thoroughly for 15 s with a low-pressure water spray according to manufacturer instructions, and completely air-dried. Then the adhesive primer (Transbond XT; 3 M-Unitek, Monrovia, CA, USA) was applied to the etched enamel and light cured for 5 s [30]. An adequate amount of composite resin (Transbond™ XT Adhesive Paste, 3 M-Unitek, Monrovia, USA) was applied to the base of the metal brackets (Roth 22 Max, Morelli, Sorocaba, SP, Brazil), which were positioned on the tooth surface with light pressure to squeeze out all the excess composite from underneath the bracket, and a dental probe was used to remove the excess composite. After accurate bracket positioning, the composite was light-cured for 40 s on the mesial, distal, incisal, and cervical sides (10 s/ side) [31] at a distance of 1 mm from the bracket base using LED curing unite with a light intensity of 400 mW/cm^2^. After light curing, the samples were stored in distilled water at 37 °C for 24 h [32]. The brackets were removed using debonding pliers.

The amount of adhesive remnants after bracket debonding was scored by three blinded examiners using the Adhesive Remnant Index (ARI) used by Bishara et al. [33]. The score ranges from 5 to 1; Score 5: meaning that there is no composite left on the enamel. Score 4: less than 10% of the composite is left on the tooth surface. Score 3: more than 10% but less than 90% of composite left on the tooth. Score 2: more than 90% of the composite is left on the tooth. Score 1: 100% of the composite left on the tooth, along with the impression of the bracket base.

### Adhesive removal and second surface roughness evaluation

**Fig. 3:**
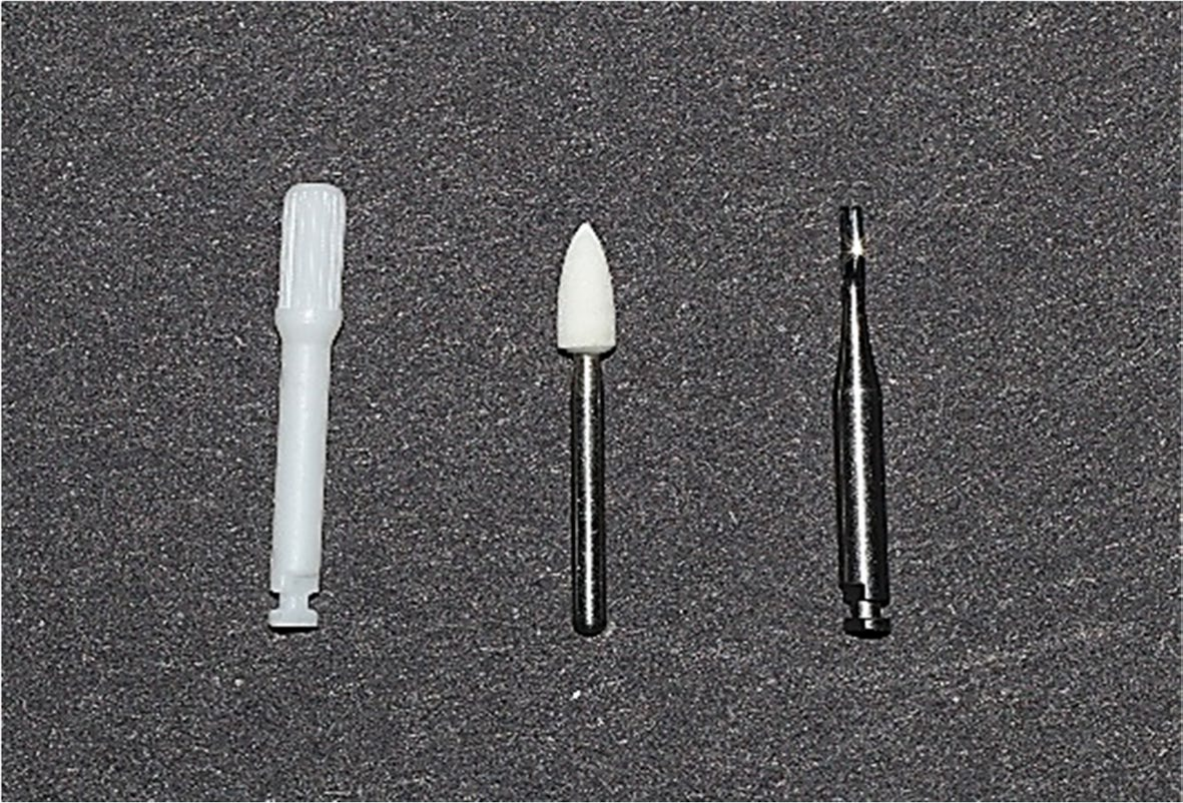
Zirconia bur, white stone bur, and tungsten carbide bur used for adhesive removal

In the first group (ZB), the adhesive remnants were removed using zirconia multiblade burs (Morelli, Sorocaba, SP, Brazil) mounted on a low-speed handpiece and used at speeds between 10.000 and 20.000 rpm. In the second group (TC), a 12-flute tapered fissure tungsten carbide bur (Hager & Meisinger GmbH, Neuss, Germany) mounted on a low-speed handpiece was used for resin remnants removal with a maximum speed of 16.000 rpm. In the third group (WS), white stone (Frank dental, Gmund, Germany) on a high-speed handpiece was used with a maximum speed of 120.000 rpm. A new bur was used for every two samples to ensure cutting efficiency and process standardization. For all the experimental groups, the clean-up process was performed according to the manufacturer’s recommendations (Fig. 3). The time required to remove the resin remnants completely from the enamel surface was recorded in seconds. The second roughness evaluation (at T1) was performed after adhesive removal, thirteen samples from each group was analyzed by profilometer and three sample from each group were examined under SEM following the same steps used in the initial roughness measurement, then Ra values and EDI scores were recorded.

### Polishing procedures and third surface roughness evaluation

After adhesive resin removal, the samples were polished sequentially with medium, fine, and super-fine Sof-Lex discs (3 M™ ESPE, Minnesota, USA) with light to moderate pressure for 15–20 s and used at different speeds, 10,000 rpm for medium grit discs and 30,000 rpm for fine and superfine grit discs with a constant, continuous, and one-directional motion to avoid creating grooves in the enamel. Final polishing was done by Sof-Lex spiral wheels, pink type (3 M™ ESPE, Minnesota, USA) on a low-speed handpiece and used at speeds between 15,000 and 20,000 rpm. For each sample, a new polishing disc and spiral were used. The surface roughness (at T2) was evaluated using the same method and steps used in previous evaluations. To reduce variability, all the procedures of clean-up and polishing were performed by the same clinician.

### Statistical analysis

Data were entered and analyzed using IBM-SPSS software (Version 26.0, 2019). Qualitative data were expressed as N (%). Quantitative data were initially tested for normality using Shapiro–Wilk’s test with data being normally distributed if *p* > 0.05. the presence of significant outliers was tested by inspecting boxplots. Quantitative data were expressed as mean ± standard deviation. The one-way ANOVA and its nonparametric alternative; Kruskal–Wallis H-test were used to compare a quantitative data between multiple groups. The two-way mixed ANOVA was used to determine whether there are differences between independent groups over time. For any of the used tests, results were considered as statistically significant if *p* value ≤ 0.05.

## Results

**Fig. 4:**
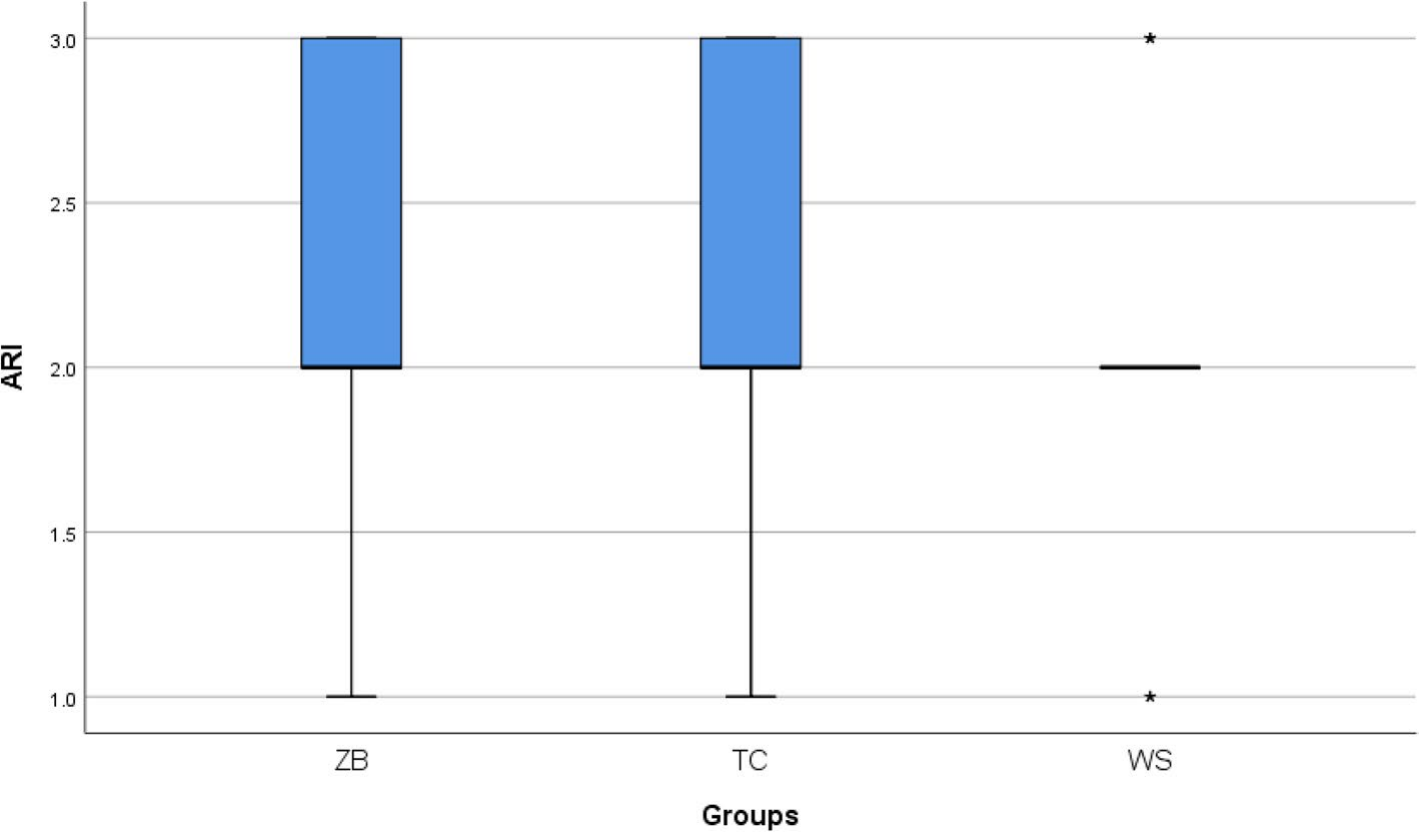
ARI in the three groups

**Fig. 5:**
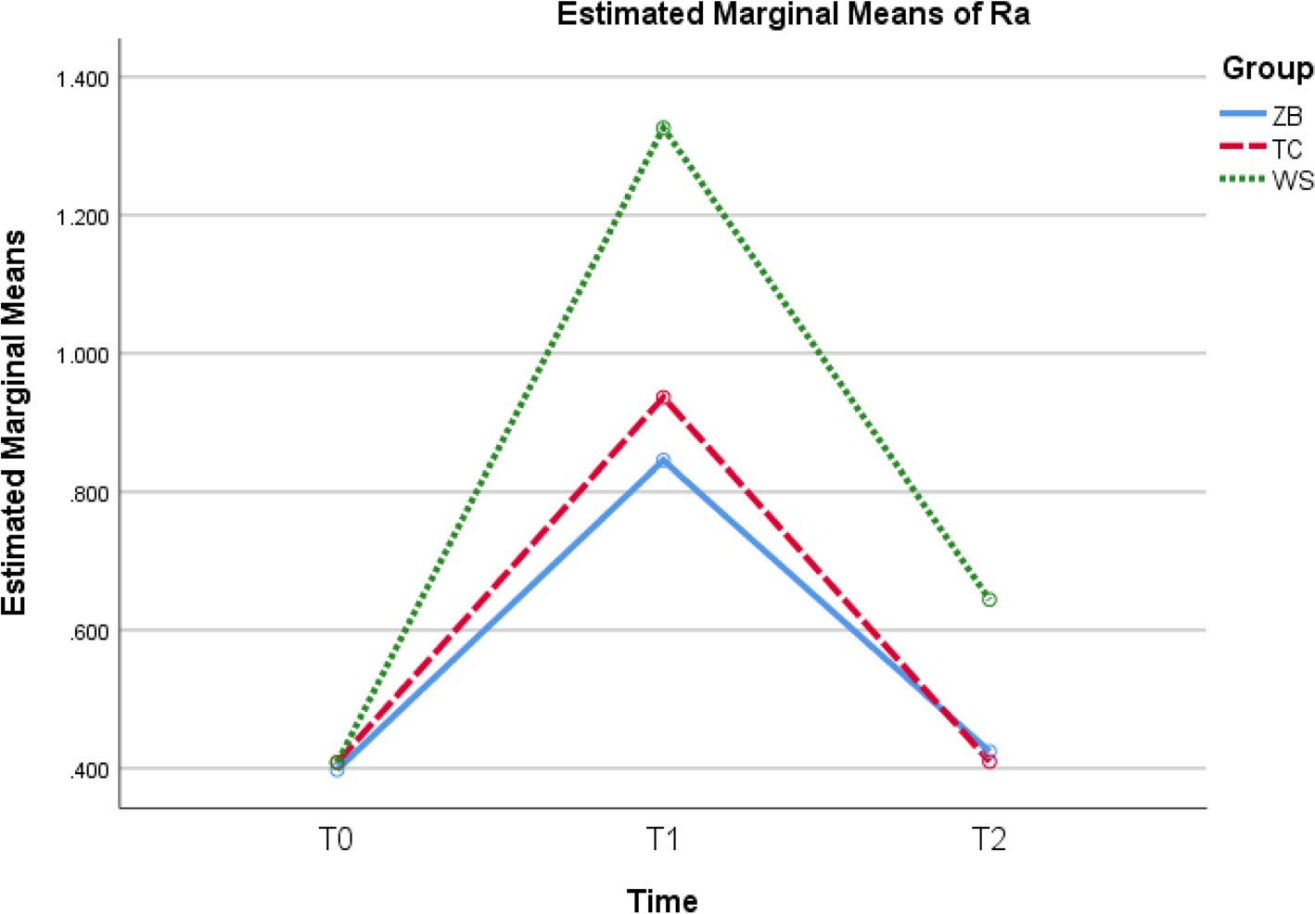
Ra values in the studied groups over time

The statistical analysis of adhesive remnants (ARI) after bracket debonding showed no statistically significant difference between all studied groups after bracket debonding (Fig. 4). Two-way mixed ANOVA revealed a statistically significant interaction between group and time on Ra values. Simple main effect for group revealed a statistically significant difference between the three groups at T1 and T2 (*p* < 0.001) but not T0 (*p* = 0.735). Pairwise comparisons at T1 and T2 revealed statistically significant difference in all pairs except ZB vs. TC at T2 (*p* = 0.377). Simple main effect for time revealed a statistically significant difference between the three time points in each group. Pairwise comparisons in each group revealed a statistically significant difference for all time pairs except T0 vs. T2 in ZB (*p* = 0.428) and TC (*p* = 1.000) (Table 1) and (Fig. 5).

**Table 1:** Two-way mixed ANOVA and pairwise comparisons of Ra among three groups at different time intervals

Group	T0	T1	T2	F	p	Partial η^2^
ZB	0.398 ± 0.043	0.845 ± 0.060	0.424 ± 0.038	90.203	** < 0.001**	0.834
TC	0.408 ± 0.038	0.936 ± 0.045	0.410 ± 0.046			
WS	0.408 ± 0.037	1.326 ± 0.070	0.644 ± 0.038			
*P֯*	0.735	** < 0.001**	** < 0.001**			
Simple main effect for group (*p*-values)						
ZB vs. TC		**0.001**	0.377			
ZB vs. WS		** < 0.001**	** < 0.001**			
TC vs. WS		** < 0.001**	** < 0.001**			
Simple main effect for time (*p*-values)						
	**ZB**	**TC**	**WS**			
T0 vs. T1	** < 0.001**	** < 0.001**	** < 0.001**			
T0 vs. T2	0.428	1.000	** < 0.001**			
T1 vs. T2	** < 0.001**	** < 0.001**	** < 0.001**			

Statistically significant at *P* < 0.05. Partial eta squared (η^2^) is a measure of effect size

*P* = parawais comparisons within each group

*P֯* = parawais comparisons within each time

EDI scores showed a statistically significant difference at T1 and T2 between WS vs. both ZB and TC with (*p* = 0.014) but not between ZB vs. TC with (*p* = 1.000), (Table 2). Regarding the time consumed for adhesive resin removal by burs, the results showed a statistically significant difference between all groups (*p* < 0.001) and Tukey’s HSD post hoc test revealed a statistically significantly longer time in the TC group > ZB group > WS group (Table 3).

**Table 2:** median and *p* value of Kruskal–Wallis H-test of EDI in 3 groups at T1 and T2

Time	ZB	TC	WS	KW-H	
				H [2]	*p*-value
T1	2 (2–2)	2 (2–2)	3 (3–3)	8	**0.018**
T2	1 (1–1)	1 (1–1)	2 (2–2)	8	**0.018**
Pairwise comparisons at T1 and T2					
ZB vs. TC					1.000
ZB vs. WS					**0.014**
TC vs. WS					**0.014**

Statistically significant at *P* < 0.05

**Table 3:** One-way ANOVA and Post-hoc Tukey HSD of Time consumed for adhesive removal (seconds) in the three groups

Statistic	ZB	TC	WS	F	*p*-value	Partial η^2^
Mean ± SD	40.23 ± 4.2	56.62 ± 4.6	33 ± 2.7	123.815	** < 0.001**	0.873
Minimum	34	49	28			
Maximum	47	64	37			
Post-hoc Tukey HSD						
ZB vs. TC					** < 0.001**	
ZB vs. WS					** < 0.001**	
TC vs. WS					** < 0.001**	

Statistically significant at *P* < 0.05. Partial eta squared (η^2^) is a measure of effect size

**Fig. 6:**
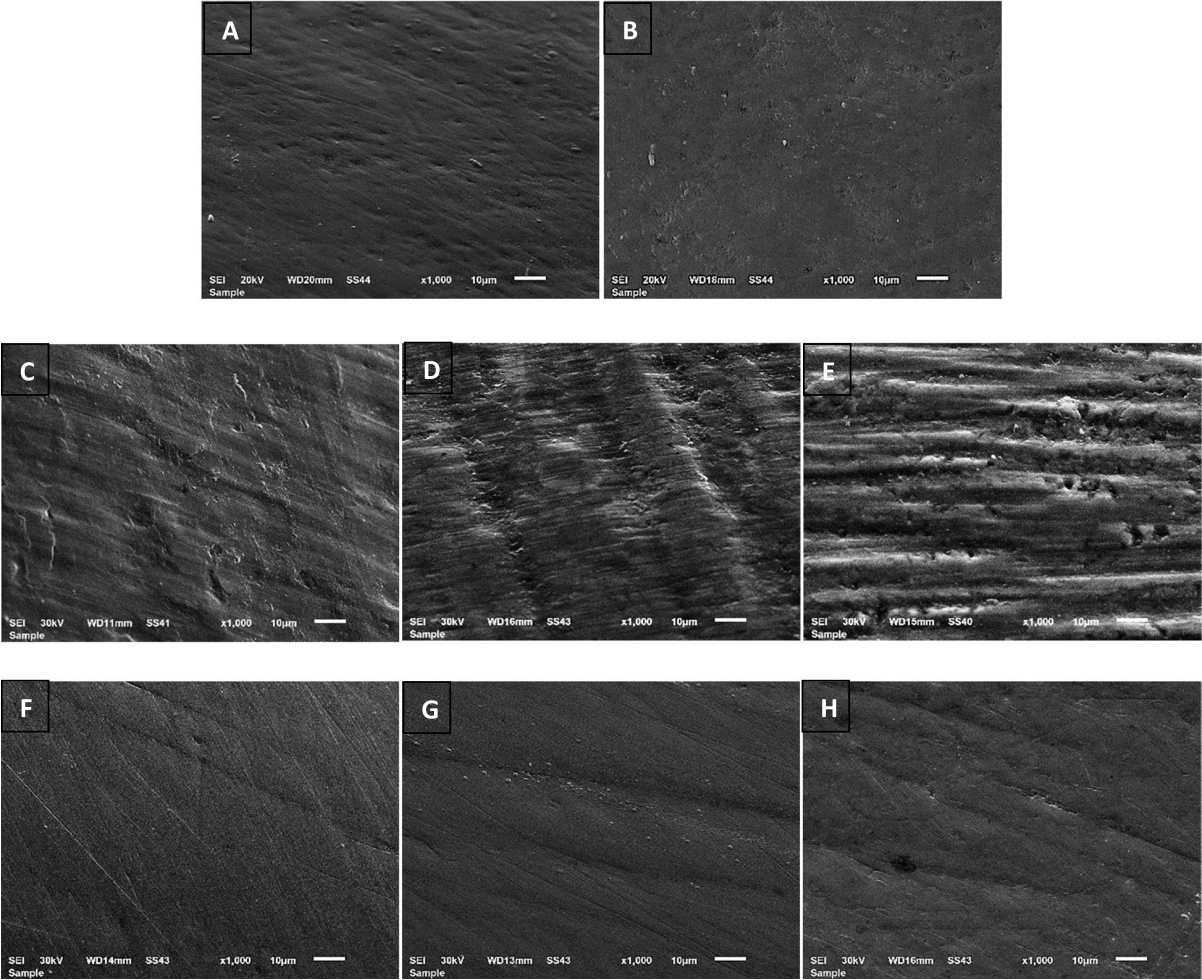
SEM photomicrographs of enamel surface at 1000X magnification. **A** and **B** enamel surface at T0. **C** enamel surface treated by zirconia bur at T1. **D** Enamel surface treated by tungsten carbide at T1. **E** enamel surface treated by white stones at T1. **F–H** enamel surface in ZB group, TC group, and WS group, respectively, at T2

The SEM images of the enamel surface at the pretreatment condition demonstrated that the enamel surface was smooth without scratches or grooves, (Fig. 6 A, B). After adhesive resin removal, ZB resulted in a roughened surface with coarse scratches and slight grooves similar to TC bur with an EDI score of 2 (Fig. 6 C, D), while WS created numerous coarse scratches, wide grooves, and enamel damage visible to the naked eye with EDI score of 3 (Fig. 6 E). The polishing using the Sof-Lex discs and spirals produced a smoother and more homogeneous enamel surface in ZB and TC groups (Fig. 6 F, G) while in WS group the enamel still had a slightly permanent damage with fewer scratches and grooves (Fig. 6 H).

## Discussion

In orthodontic treatments that use fixed appliances or clear aligners, the brackets or attachments are bonded to enamel and when the orthodontic treatment is over, a major challenge is the removal of those attachments and adhesive resin from enamel surface with less damage to the enamel surface as possible. The removal of superficial enamel might make the enamel less resistant to the organic acids in the oral environment, which can increase the risk of demineralization [22]. Many techniques were introduced over the years to remove the adhesive remnants after bracket debonding with less surface roughness and less enamel damage. However, no standard, approved technique that can direct the orthodontist in their clinical practice to the ideal method [34].zirconia bur is designed to remove adhesive resin from the enamel surface after bracket debonding. According to the manufacturer they are very resistant to fracture and has eighteen blades and a rounded tip to reduce the gingival damage. This in vitro study aimed to compare the effects of different burs; ZB, TC, and WS on enamel surface roughness. Also, the time consumed for adhesive removal was recorded in seconds.

In this study, ARI scores showed no statistically significant difference between all the groups as regards the amount of adhesive remnants left on the tooth surface after bracket debonding, with a predominance of scores 2 which was similar to Vidor et al*.* [35] study.

In this study, Ra values and SEM images showed that WS resulted in greatest surface roughness and enamel damage as compared to TC. These results agree with Eliades et al*.* [12] who observed that the carbide bur was more effective for adhesive removal and resulted in less enamel loss. Sugsompian et al. [36] and Janiszewska-Olszowska et al*.* [37] also demonstrated that the tungsten carbide bur produced lesser surface roughness than white stones, and they recommended not to use WS for adhesive removal as they cause severe irreversible enamel damage. As opposed to our results, Mohebi et al*.* [38] found no significant differences in surface roughness between WS and TC bur in their studies. This controversy may be attributed to that they used WS in a low-speed handpiece. Also, Gwinnett and Gorlick [10], Ulusoy [8], Cesur et al*.* [17], and *Karan *et al*.* [15] revealed that TC bur resulted in sever enamel damage and increased surface roughness as compared to other methods especially when used on high-speed handpiece.

The current study showed that the ZB was efficient at removing the adhesive remnants after orthodontic debonding and created a lower surface roughness than TC burs and WS. SEM microphotographs showed that the enamel surface topography in the ZB group was similar to that in the TC group, with numerous coarse scratches and slight grooves. Therefore, according to the obtained results, we recommend using ZB for adhesive removal as they are effective for adhesive removal with little enamel damage and after final polishing, they leave a smooth tooth surface that decreases the chances of plaque accumulation and subsequent enamel decalcification and discoloration. Also, they are a moderately time-consuming method compared to TC burs, which consumed more time.

The same polishing protocol was applied for all groups using Sof-Lex discs and Sof-Lex spirals. The enamel surface was restored approximately to its pretreatment condition after polishing in samples treated with ZB and TC burs, while Ra decreased considerably in WS groups but still had a slightly roughened surface with irreversible fewer scratches and shallow grooves after polishing. This is consistent with the results concluded by Ozer et al. [26] and Pinzan-Vercelino et al. [39] who reported that the use of Sof-Lex discs and Sof-Lex spirals for polishing has restored the enamel surface closer to its pretreatment condition with less enamel surface damage. Also, a systematic review by Janiszewska-Olszowska et al. [37] concluded that the enamel surface must be sequentially polished with the Sof-Lex discs and Sof-Lex spirals for being the most reliable polishing method. Howell and Weeks, [40] disagreed with these results and concluded that the medium and fine Sof-Lex discs produced the roughest tooth surface during polishing. This disagreement might be because the Sof-Lex discs were used in a dry condition.

Regarding the time required for adhesive resin removal by the different evaluated burs, there were statistically significant differences between all groups. The WS was the least time-consuming method with an average time (33.0 ± 2.7 s) followed by the ZB (40.2 ± 4.2 s). However, the TC was the more time-consuming method (56.6 ± 4.6 s). Similar results of Tenório et al*.* [27] have shown that the time spent for resin remnants removal by the tungsten carbide bur was 56 ± 5.2 s. *Shafiee *et al*.* [41] had conflicting results, stating that faster adhesive removal was achieved by using the TC bur as compared to WS, this disagreement may be because they used WS on a low-speed handpiece. Also, *ulusoy* [8] concluded that the TC bur was the least time-consuming method and these conflicting results might be due that they used high-speed TC burs.

### Limitations of this study

Our study has some limitations that should be addressed in future research. This in vitro study could not mimic the oral environment as intraoral factors such as saliva, oral hygiene, temperature, and pH were not considered [8]. Also, advanced evaluation methods, such as confocal laser microscopy and atomic force microscopy (AFM), could be used in future studies to obtain 3D data on enamel roughness and precise information about the amount of enamel loss caused by various resin removal methods.

## Conclusion

Within the limitations of this in vitro study, we concluded that; 1) The null hypothesis of insignificant difference in the quality of enamel surface roughness following orthodontic deboning in tested groups was rejected. 2) the ZB was effective for adhesive resin removal and produced the lowest surface roughness and the least enamel damage, so it can be considered an alternative approach with a moderately time-consuming process. 3) The TC bur produced comparable results to the ZB but was more time-consuming. 4) The WS created the greatest surface roughness and irreversible enamel damage, although they were the least time-consuming method. 5) Polishing is a necessary step after adhesive removal by different burs.

## Data Availability

All data generated or analyzed during this study are included in this published article.

## Abbreviations

μm: Micrometer
ARI: Adhesive Remnant Index
EDI: Enamel Damage Index
Er: YAG: Erbium: Yttrium–Aluminum-Garnet Laser
HMDS: Hexamethyldisilazane
KV: Kilovolt
LED: Light Emitting Diode
mA: Milliampere
mm: Millimeter
mW: Milliwatt
n: Sample size
Ra: Surface roughness
rpm: Revolutions per minute
SEM: Scanning Electron Microscopy
T0: Time before bracket bonding
T1: Time after adhesive resin removal
T2: Time after polishing
TC: Tungsten carbide bur group
UV: Ultraviolet light
WS: White stone group
ZB: Zirconia bur group
